# Advanced Practice Clinician Training for Neurology

**DOI:** 10.7759/cureus.1196

**Published:** 2017-04-26

**Authors:** David M Ermak, Lori Cox, Aiesha Ahmed

**Affiliations:** 1 Department of Neurology, Penn State Hershey Medical Center; 2 Department of Neurosurgery, Penn State Hershey Medical Center

**Keywords:** neurology, training, education, physician assistant, medical practitioners

## Abstract

**Background:**

The specialty of Neurology is faced with a fundamental problem of economics: supply and demand. The projected increase in provider supply is unlikely to keep up with projected increases in patient-care demand. Many large academic centers have used residents to meet this patient-care demand. However, the conflict between education of residents and patient-care needs has created a hindrance to both of those missions. Many specialties have been using advanced practice clinicians (APCs) to help address the need for patient care. In the setting of a residency program, this availability of APCs can help to alleviate patient-care demands for the resident and allow for better allocated educational time. Neurology has not historically been a popular choice for APCs and a standardized educational curriculum for a Neurology APC has not been established.

**Methods:**

The authors share an example curriculum recently implemented for training new inpatient Neurology APCs. This curriculum includes a 12-week program complete with rotations through various subspecialties and proposes fundamental lecture topics for use in education. The authors share their expectations for clinical duties that evolve over the course of the 12-week program in conjunction with expectations for increasing clinical knowledge as well as efficiency in system utilization.

**Conclusion:**

The addition of APCs to support a busy inpatient Neurology practice has obvious beneficial implications but the integration and education of this new staff must be structured and well-designed to support the confidence of the APC in both their knowledge and their role as an indispensable member of the care team.

## Introduction

The current number of US neurologists is considered unacceptable in view of the increase in age-related neurologic diseases in the population. Therefore, many large academic medical centers have relied on neurology trainees such as residents and fellows to help with meeting the needs of the population. However, this has created a conflict between education and patient care responsibilities whereby the clinical workload of a resident interferes with the educational requirements mandated during residency [1]. This concern can be best addressed by the addition of well-trained advanced practice clinicians (APCs) who can offset the clinical workload. In this context, APCs include physician assistants (PA) and certified registered nurse practitioners (CRNP); both of whom have varying degrees of autonomy in each state but will be collectively referred to as APCs in this article. This strategy can be further supported by the fact that the estimated active supply of 16,366 US neurologists in 2012 is projected to increase to 18,060 by 2025. Demand for neurologists is projected to increase from ∼18,180 in 2012 (11% shortfall) to 21,440 by 2025 (19% shortfall) [2]. Therefore, the utilization of APCs trained in the essentials of neurologic diagnosis and management offers a potential solution to the growing demand for this specialty.

In many contexts, APCs are already involved in the provision of neurological services. The American Academy of Neurology (AAN) reported that APCs were employed in 39% of neurology practices with a higher percentage of them used in the hospital setting [3]. Taft and Hooker performed a survey of 46 PAs who worked full-time in a neurology practice. The inpatient-based APCs saw approximately 26 patients per week and outpatient-based APCs reported an average of 43 patients a week; most APCs worked a 40-hour week. The most common conditions managed by APCs in neurology included headaches, stroke, Parkinson’s disease, epilepsy, and multiple sclerosis. Headaches were the most frequently seen diagnoses. In this study, the APCs reported that the services they provided were similar to the neurology physicians. APCs also spent a significant amount of time on preventative care in the form of patient and family counseling and education. About two-thirds cited lumbar punctures as a procedure performed by them without supervision. About 50% reported that they are involved in research studies, serving as coordinators, data collectors, or examiners. The surveyed APCs noted a direct role in helping with decreasing patient waiting times [4].

Neurology is a satisfying career selection for APCs according to the AAN survey; job satisfaction was noted to be high with low turnover rates coupled with average time working in neurology being more than seven years [4]. The above-noted information points out that most APCs enjoy reasonable working hours with an exposure to a variety of clinical and non-clinical activities which can be considered useful from a professional development standpoint. Also, the ability to get involved with multiple different subspecialty patients, procedure, and non-clinical academic activities may lead to higher job satisfaction and job retention.

Current APC education may not involve adequate exposure to neurology to ensure that high-quality patient care can be provided without changes to the traditional curriculum. The Accreditation Review Commission on Education for the Physician Assistant, Inc. (ARC-PA) requires the following areas for the preclinical curriculum: anatomy, physiology, pathophysiology, pharmacology, and genetic and molecular mechanisms of disease. In the clinical curriculum, the required areas are emergency medicine, family medicine, general internal medicine, general surgical care, geriatrics, pediatrics, prenatal care, and women’s health. Training programs for advanced practice nursing are far more variable as there are numerous types of programs available. Historically, education for APNs has been described as “uncoordinated and sporadic, resulting in programs that have been inconsistent in academic preparation, duration, theory base, and practice requirements” [5].

There are a number of specialty post-graduation programs for PAs, but only a single program for neurology exists (Table 1) [6]. The variable exposure of APCs to neuroscience and neurology in the preclinical and clinical curricula along with the paucity of available post-graduate training gives rise to valid concerns regarding the general competency level of APCs in clinical neurological care. A parallel exists in the training of MDs and DOs who may or may not be required to complete a Neurology clerkship for graduation depending on the regulations of their medical school, underscoring the critical importance of formal residency training with its >10,000 hours of patient care and study in a given specialty as a practical prerequisite for independent practice. Given these concerns and the expanding clinical need, we developed a specialized training program for neurology APCs as described here.

**Table 1:** Distribution and Enrollment of Postgraduate Physician Assistant Training Programs in 16 Medical and Surgical Specialties, 2007* *This table was reproduced with permission from the authors [6].

Specialty	No. of Programs	Total Annual Enrollment
Cardiothoracic surgery	3	3-5
Critical care	1	2-5
Dermatology	1	2
Emergency medicine	5	10-15
Hospitalist	2	13
Neonatology	1	2
Neurology	1	2
Neuroradiology and sleep medicine	1	1-2
Neurosurgery	1	1
Obstetrics-gynecology	1	4-6
Oncology	1	2
Orthopedic surgery	5	10-16
Psychiatry	2	5
Rheumatology	1	1-2
Surgery	12	44-48
Trauma	2	3
Urology	1	2
Total	41	106-116

## Materials and methods

Our hospital is an academic medical center with 551 licensed beds, over 29,000 admissions per year with over 960,000 emergency room visits per year. We have about 1500 inpatient neurology admissions per year and about 20,000 outpatient neurology visits per year. Our department has been utilizing APCs in the outpatient setting for over a decade. We had expanded our outpatient APC employment over the last two years with a large focus on improving access to Neurology. This expansion along with the development of an access clinic was successful in reducing lead time for new patient visits from about one year down to several weeks [7].

Given our intradepartmental success with the use of APCs, and faced with a growing inpatient census, we saw an opportunity to incorporate APCs into the inpatient setting to decompress the workload on our residency program. The APC would work alongside the residents and perform history and physicals, write orders, complete pre-rounding, write progress notes, prepare discharge summaries, and address any urgent patient issues. Our inpatient service line is separated in two teams, one dedicated to stroke and the other to general neurology; each typically comprised of two residents and one attending. The APCs would ultimately alternate between the two service lines every four weeks, expanding each team to at least four providers.

Our approach to curriculum development was to integrate two APCs into our inpatient neurology program which was already comprised of 12 residents (four per post graduate year). The orientation and training program was designed by a team of providers from within the neuroscience group at our hospital. This team was comprised of physicians and APCs representing both Neurology and Neurosurgery. The premise of creating such a curriculum was to ensure that our APCs would be given the basic fundamentals to practice neurology in a supervised capacity. In our outpatient experience, we had experienced difficulties in competency which was felt to be related to lack of a standardized educational curriculum.

We created an orientation and training program for this position that would take place over 12 weeks. The APC will rotate through all necessary aspects of the service to provide adequate introductory exposure. These aspects included Stroke Service, General Service, Epilepsy Monitoring Unit, Outpatient Neurology Clinic, Neurophysiology Lab, Interventional Neuroradiology, and Acute Inpatient Rehab. At the start of orientation, the APC is exposed to a Neurologic Examination tutorial video and in person training. During each day, in addition to clinical responsibilities, there is some combination of training, certification, focused lectures, and reference articles for review. Pertinent clinical certification and training included National Institutes of Health Stroke Scale (NIHSS), Modified Rankin Scale (MRS), and brain death evaluation. Since there is a Neurology residency at our facility, we encourage the APC to attend the daily residency lectures ad-lib. We developed additional targeted lectures for introductions to all conditions and diseases that are frequently encountered on our inpatient service. A list of topics by week can be found in Table 2. A comprehensive calendar was built with word-processing software which contained links to reference articles, websites, and other online training material; this calendar was provided electronically so that access to all resources was immediately available.

**Table 2:** Topics for Lecture, Independent Reading, and Certification Across the Orientation Timeline AED: Antiepileptic drug; CT: Computed tomography; EHR: Electronic health record; ICH: Intracerebral hemorrhage; ICP: Intracranial pressure; IRB: Internal review board; IV tPA: Intravenous tissue plasminogen activator; MRI: Magnetic resonance imaging; MRS: Modified Rankin Score; NIHSS: National Institutes of Health Stroke Scale; SAH: Subarachnoid hemorrhage.

Timeframe	Lecture Topics	Independent Reading/Certification
Week 1	None	EHR training
Week 2	The complete neurologic examination; cerebrovascular anatomy; pathophysiology of atherosclerosis	NIHSS certification; MRS certification; IV tPA indications and use
Week 3	Acute stroke management; secondary stroke prevention; cardiac causes of stroke	Guidelines for primary and secondary prevention of ischemic stroke; guidelines for management of ICH
Week 4	Neuroimaging with CT and MRI; stroke core measures; telemedicine for stroke care	Guidelines for SAH management; endovascular therapy for ischemic stroke
Week 5	Brain death	IRB training
Week 6	Critical care management of stroke	Management of elevated ICP
Week 7	Altered mental status; substance abuse; meningitis; headache	Management of migraine headache; evaluation of altered mental status
Week 8	Peripheral neuropathy; acute non-traumatic weakness	Guillain-Barre syndrome; myasthenia gravis; transverse myelitis
Week 9	Epilepsy management; status epilepticus management; pharmacology of AEDs	Guidelines for status epilepticus; management of drug resistant epilepsy; vagal nerve stimulation for epilepsy
Week 10	Dementia; Parkinson’s disease; drug-induced movement disorders	Deep brain stimulation
Week 11	Multiple sclerosis; palliative care in neurology	Metabolic causes of encephalitis
Week 12	Muscular dystrophy; myopathy	Muscle disease

A detailed timeline of the clinical duties for the entire 12 weeks is provided in Table 3. During these 12 weeks, there is an expected acquisition of knowledge of skill that should be taking place through clinical exposure, teaching on rounds, reading, and attending lectures. A summation of this expected progress is found in Table 4.

**Table 3:** Clinical Duties of the APC APC: Advanced practice clinician; EEG: Electroencephalography; EMG: Electromyography.

Timeframe	Rotation	Clinical Duties
Week 1	Hospital orientation	None
Week 2	Stroke service	Shadowing the stroke team; observing the work-flow, and becoming exposed to the patient population
Week 3	Stroke service	APC begins to take ownership of patients and participates in daily duties of pre-rounding and note writing; all decisions are discussed directly with the attending physician in real-time
Weeks 4-6	Stroke service	Increasing patient load is put onto the APC with continued direct supervision. Several days are spent in neurointerventional radiology and acute inpatient rehab
Week 7	Outpatient exposure	APC shadows in general neurology clinic and neurophysiology lab where they are exposed to EEG and EMG
Week 8	Epilepsy monitoring unit (EMU)	Admits patients to the EMU and follows them daily through discharge. Reads EEG with attending
Weeks 9-12	General neurology	The APC returns to the inpatient service and is involved in direct patient care for patients on the general neurology service. Direct supervision from the attending is continued but the APC is expected to take on an increasing patient load as increasing competence is demonstrated and should begin to show efficiency in daily work

**Table 4:** Expected Progress of the APC APC: Advanced practice clinician; CT: Computed tomography; EEG: Electroencephalography; EHR: Electronic health record; IV tPA: Intravenous tissue plasminogen activator; MRI: Magnetic resonance imaging; MRS: Modified Rankin Score; NIHSS: National Institutes of Health Stroke Scale.

Timeframe	Expected Progress
Week 2	Become familiar with the diagnosis of stroke and the pathophysiology behind the disease process. Learn basic vascular neuroanatomy
Week 3	Learn the basics of secondary stroke prevention and medical management of the disease. Be able to perform an NIHSS and calculate an MRS
Week 4	Become familiar with neuroimaging modalities of CT and MRI. Become efficient at the use of the EHR
Week 5	Learn to manage the acute stroke patient and become familiar with the use of IV tPA and other intra-arterial procedures
Week 6	Feel confident in the day-to-day management of common issues in the stroke population (e.g., blood pressure management)
Week 7	Appreciate the vast array of the spectrum of neurologic disease and become familiar with the abilities and limitations of outpatient disease management
Week 8	Become familiar with a normal EEG tracing as well as learn about the common antiepileptic drugs
Week 9	Become exposed to common inpatient neurologic diagnoses such as Multiple Sclerosis, headache, Guillan-Barre Syndrome, encephalopathy, etc.
Week 10	Learn the management strategies for the commonest inpatient neurologic disorders and be exposed to lumbar punctures
Week 11	Feel confident in performing a complete neurological examination and be able to provide thoughtful differential diagnosis
Week 12	Become proficient at the daily tasks of pre-rounding, progress note writing, admitting, discharging, and EHR use

During the orientation timeframe, routine short written multiple choice tests were given corresponding to the teaching and reading being assigned that week. Each test was 10 questions long and test topics included stroke, headache, epilepsy, neuromuscular disease, and two tests on other general neurology. Testing was used to assess acquisition and integration of clinical knowledge. Testing was reviewed in person between the APC and the Neurologist responsible for the orientation. When knowledge gaps were identified, additional resources were dedicated to close said gaps. At the conclusion of the orientation process, the APC no longer had any required additional lectures to attend but was invited to continue to attend any and all Neurology resident lectures that were being given.

Identifying the sources to procure the most salient and reliable information can be difficult. We recommend using the most up to date guideline statements where applicable (e.g., American Stroke Association, American Academy of Neurology, etc.). Internally cultivated lectures are indispensable for transmission of knowledge and also help the APC to learn the preferences of their colleagues when it comes to subtle variations in practice. Where guideline statements and lectures don’t suffice it is reasonable to provide references to high-quality literature that focus on evidence-based medicine.

## Results

No formal evaluation process took place but an ongoing dialogue was kept between the primary supervisor and the APC during 12 weeks of the orientation and training program. The most well-liked parts of the program were reported to be the detailed calendar of articles to review and references for additional training and certification and the lecture series. The two APCs who have completed our program believe the training to have been valuable both from an educational standpoint but also from an operational standpoint; allowing time for a new hire to adjust to the work-flow and intricacies of a new hospital system resulted in satisfaction amongst the APCs who felt better equipped to function as an independent member of the team at the completion of orientation. From the supervisor’s perspective, the periodic short knowledge-based testing was valuable to identify potential shortcomings in the educational process. Both APCs who completed the program demonstrated excellent acquisition of knowledge as assessed by this testing during the orientation process. However, both APCs scored poorly on testing related to dementia by comparison to other topics.

## Discussion

In our opinion, teaching on rounds is crucial to the cultivation of knowledge not only during the orientation but also across the continuum of employment. However, we believe that bedside teaching alone cannot effectively cultivate a well-rounded APC and therefore it is our opinion that having a structured schedule with focused high yield topics is imperative to this orientation process.

In any teaching scenario, feedback plays a critical role in the learning process. Feedback was given by the supervising physician; this person was involved in the screening and hiring process, and was identified as the point of contact for the APC. We sought to provide feedback via face-to-face meetings every two weeks. These meetings were kept informal and brief, but allowed for the APC to provide feedback on such things as clinical load, value of lectures, teaching on rounds, and overall experience. Prior to the meetings feedback was collected from residents, attending physicians, and other staff members on any thoughts or concerns they had about the APC.

Supervision and oversight of the APC training process was a dualistic approach with a formally designated supervisor as mentioned above providing remote oversight, while each attending on service provided direct supervision and oversight on a day to day and patient to patient basis. The APC was granted variable levels of independence based on the comfort level of both the attending physician and the APC. This independence is heavily influenced by patient complexity and APC experience; at any point, the attending should be available to provide direct oversight but if both the attending and the APC agree, then the patient can be seen by the APC alone.

To date, we have had two APCs complete this training process which is a limitation in our ability to fully evaluate the merits of such a training program. Furthermore, since the inpatient APC position is new for our department, we do not have any comparison of an APC who did not complete this type of curriculum training. As more APCs complete our program we will plan to gather their feedback on the efficacy of this curriculum in order to continue to modify and update our program to reflect any shortcomings of our current structure. One of the shortcomings that has already been identified is related to the relatively low scores on testing related to the topic of dementia. In part, this can be explained by the virtue of dementia being largely an outpatient-related condition but also our program currently lacks a specialist in Cognitive and Behavioural Neurology. Due to these relatively low scores, we have added additional reference material and lectures on this topic to enhance the knowledge in this area.

## Conclusions

Standardizing an orientation and training program for new hire APCs can ensure that each individual is provided with a similar foundation of knowledge and skills such that integration into a subspecialty, such as neurology, can be accomplished despite the overall lack of post-graduate training for APCs. It is our experience that the APCs find this time period focused on education and training to be a valuable and positive experience. In our institution, we are now exploring a partnership with our affiliated Physician Assistant School to provide clinical experience in Neurology during the clinical phase of the degree program to enhance the pre-graduate exposure to Neurology as a subspecialty.
